# Post-stroke pain syndromes in Africa: epidemiology, clinical phenotypes, and unmet needs in assessment and management

**DOI:** 10.3389/fneur.2026.1946268

**Published:** 2026-09-22

**Authors:** Nicholas Aderinto, Thomas Oyediran, Temitomi Jane Oyedele, Nwamaka Chidera Bob-Ume

**Affiliations:** 1 Department of Medicine, Ladoke Akintola University of Technology, Ogbomoso, Nigeria; 2 College of Health, Ladoke Akintola University of Technology, Ogbomoso, Nigeria; 3 Department of Medicine and Surgery, Bowen University, Iwo, Osun, Nigeria; 4 School of Public Health, University of Port Harcourt, Port Harcourt, Nigeria

**Keywords:** Africa, pain, post-stroke pain, stroke, survival

## Abstract

Stroke disproportionately affects Africa, where incidence and prevalence rates rank among the highest reported worldwide, and case-fatality remains high. As acute survival improves in many, though not all, African settings, the burden of chronic post-stroke sequelae is growing, yet non-motor complications such as pain remain comparatively neglected in both clinical practice and research relative to motor and cognitive outcomes. Post-stroke pain is a heterogeneous group of syndromes, including central post-stroke pain, hemiplegic shoulder pain, spasticity-related musculoskeletal pain, post-stroke headache, and complex regional pain syndrome, each with distinct mechanisms and management pathways. This narrative review summarizes the sparse but growing African evidence base on post-stroke pain epidemiology and clinical phenotypes, drawing on data from Egypt, Nigeria, Zimbabwe, Ghana, Kenya, South Africa, and Uganda, and situates it within global comparators. We identify substantial unmet needs across assessment, including the absence of pain-screening tools validated in African languages, limited diagnostic infrastructure, and low clinician awareness, and across management, including restricted pharmacological access, scarce rehabilitation workforce, limited access to interventional options, and the parallel, poorly integrated use of traditional and complementary medicine. We propose a research and policy agenda prioritizing validated assessment tools, phenotype-specific prevalence studies, and pragmatic, resource-appropriate management models suited to African health systems.

## 1 Introduction

Stroke is the world’s second leading cause of death and a leading combined cause of death and disability, with nearly 87% of stroke-related deaths and 89% of disability-adjusted life-years lost occurring in low- and middle-income countries (1). Within this global context, Africa bears a disproportionate stroke burden. Across African studies, incidence has reached 316 per 100,000 person-years, prevalence 1,460 per 100,000 in a Nigerian population, and three-year case-fatality over 80%, although estimates vary substantially across settings and study populations (2). Age-adjusted prevalence in some African settings now exceeds global averages even as prevalence declines in Western Europe and North America (3). Community and hospital-based surveys illustrate wide variation across the continent, from a crude prevalence of 58 per 100,000 in rural southwestern Nigeria to 963 per 100,000 in urban Egypt, and from an incidence of roughly 14 events per 1,000 person-years in Ghana to considerably higher rates in other settings (3, 4). Within Nigeria alone, prevalence estimates across different regions range from 0.36% to over 1.3% depending on the population and methodology used (5).

Hypertension remains the dominant modifiable risk factor across African populations, and strokes frequently occur one to two decades earlier in life than in high-income settings, amplifying the socioeconomic and caregiving impact on survivors, families, and health systems (2, 3). As acute-phase mortality gradually improves with expanding, if still limited, access to hospital-based stroke care, the population of long-term survivors living with chronic post-stroke sequelae continues to grow. Non-motor complications of stroke, however, have historically received far less clinical and research attention in Africa than motor recovery, cognitive impairment, or mortality. Post-stroke depression, for example, remains under-recognized despite systematic review evidence confirming it as a frequent and burdensome complication across African cohorts (6), and pooled estimates from meta-analyses spanning Africa alongside West and South Asia report substantial prevalence of both post-stroke depression and anxiety, alongside a call for more culturally sensitive assessment tools tailored to these populations (7).

Post-stroke pain follows this same pattern of relative neglect, despite being common, functionally disabling, and a direct impediment to rehabilitation participation. Internationally, post-stroke pain is understood not as a single condition but as a heterogeneous family of syndromes with distinct underlying mechanisms: central post-stroke pain arising from central sensory pathway injury, hemiplegic shoulder pain arising from a combination of biomechanical, neurogenic, and soft-tissue causes, spasticity-related musculoskeletal pain, post-stroke headache, and complex regional pain syndrome affecting the hemiplegic limb. Each phenotype carries distinct diagnostic criteria, natural history, and treatment approach. In African settings, the available evidence on these syndromes is fragmented across a small number of single-center studies concentrated in a handful of countries, embedded as secondary findings within broader stroke-outcome or caregiver-burden research, or entirely absent for some phenotypes and regions. This narrative review synthesizes available African evidence on the epidemiology and clinical phenotypes of post-stroke pain, situates it against the global evidence base, and identifies the unmet needs in assessment and management that should shape research and health-system priorities for the region Figure 1.

**Figure 1:**
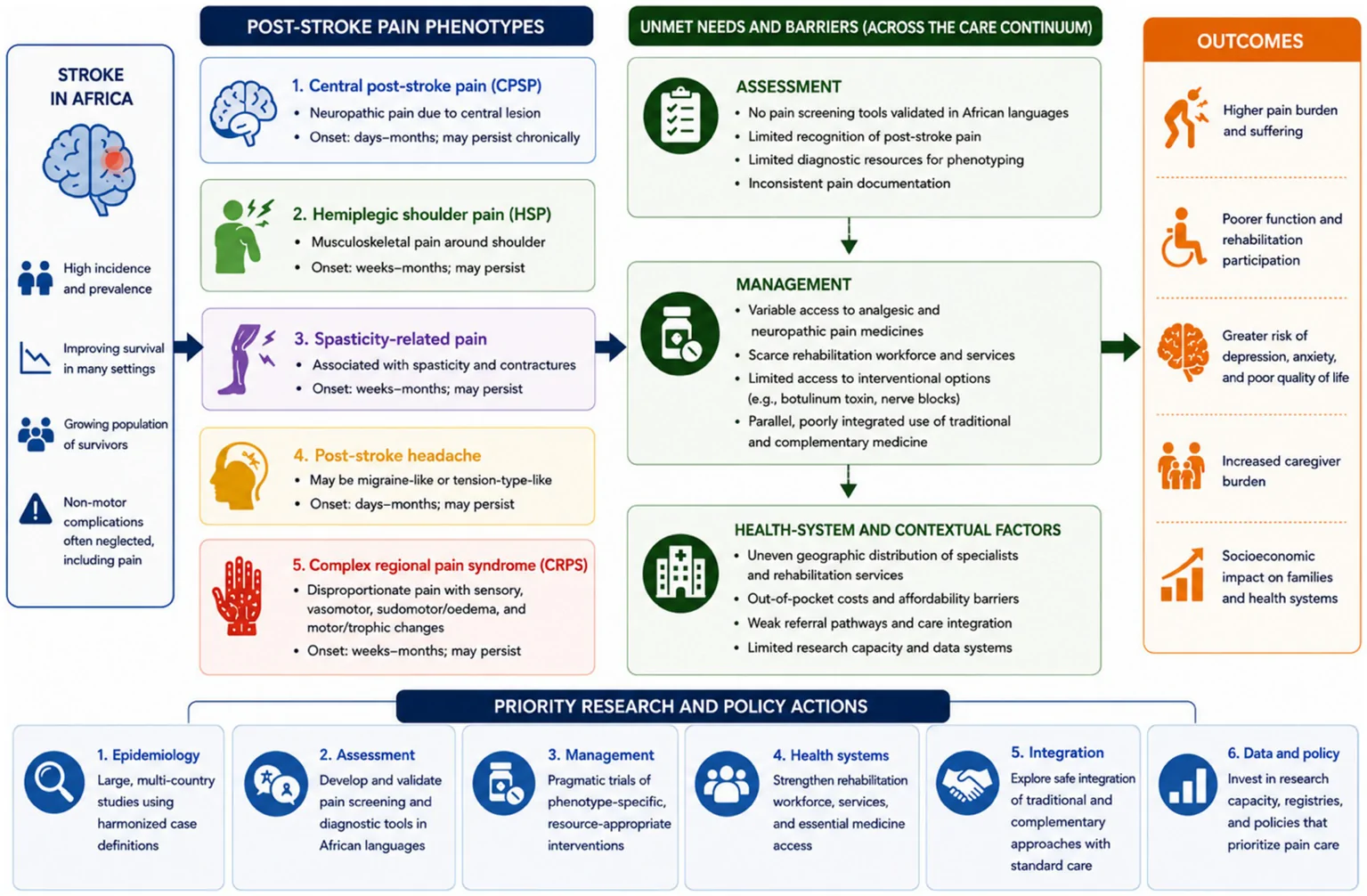
Conceptual figure for the paper.

## 2 Methodology

This narrative review used a structured database search but was not designed as a systematic or scoping review. We searched PubMed, African Journals Online, Embase, Web of Science, and Scopus from inception to June 2026. Search terms combined stroke-related terms with post-stroke pain phenotypes, including central post-stroke pain, neuropathic pain, hemiplegic shoulder pain, spasticity-related pain, headache, complex regional pain syndrome, and shoulder-hand syndrome, together with geographic terms for Africa and individual African countries. Boolean operators and database-specific controlled vocabulary and field tags (e.g., MeSH and Emtree) were used where applicable. No language filter was applied; however, only studies with an available English-language full text were assessed.

Two authors (NA and TO) independently screened titles, abstracts, and full texts, with disagreements resolved by discussion or consultation with a third author (TJO). Eligible studies reported survivor-level data on defined post-stroke pain phenotypes in adults (≥18 years) following ischemic stroke, intracerebral hemorrhage, or subarachnoid hemorrhage in an African country. Caregiver pain, studies without survivor-level pain outcomes, non-empirical reports, and traditional-medicine studies without pain outcomes were excluded from the core synthesis and, where relevant, considered separately as contextual evidence. Reference lists of included studies and relevant reviews were also hand-searched. No formal risk-of-bias assessment was undertaken.

## 3 Epidemiology of post-stroke pain in Africa

Direct epidemiological data on post-stroke pain in Africa are sparse relative to the volume of literature on stroke incidence, risk factors, and mortality on the continent, but a small cluster of dedicated central post-stroke pain (CPSP) studies now spans three countries with strikingly different prevalence estimates. The highest reported figure comes from Egypt, where a prospective study of 65 consecutive ischemic stroke patients at Suez Canal University Hospitals found CPSP in 35.4% (23/65), with deep sensory dysfunction, thalamic stroke location, smoking history, and age under 50 years identified as significant predictors using somatosensory evoked potentials and MRI correlation (8). This contrasts with lower estimates from West and Southern Africa. A cross-sectional study of 120 stroke survivors in Nigeria reported CPSP in 5% (6/120), with onset within 3 months in half of cases (9). Although the study later describes sensory characteristics in 11 patients with CPSP, creating an internal inconsistency in the reported denominator, the 6/120 prevalence estimate is retained here, and the subgroup-derived percentages are not used. In Zimbabwe, a study of 166 stroke survivors reported an 8% prevalence of CPSP, with younger age (<60 years) associated with CPSP and hyperaesthesia, electric-shock-like pain, and thermal and mechanical allodynia among the reported features (10). Health professional practices captured in the Zimbabwean study were informative from an assessment-gap perspective: only a small minority of surveyed health professionals reported using any validated neuropathic pain screening instrument in routine practice (10) Figure 2.

**Figure 2:**
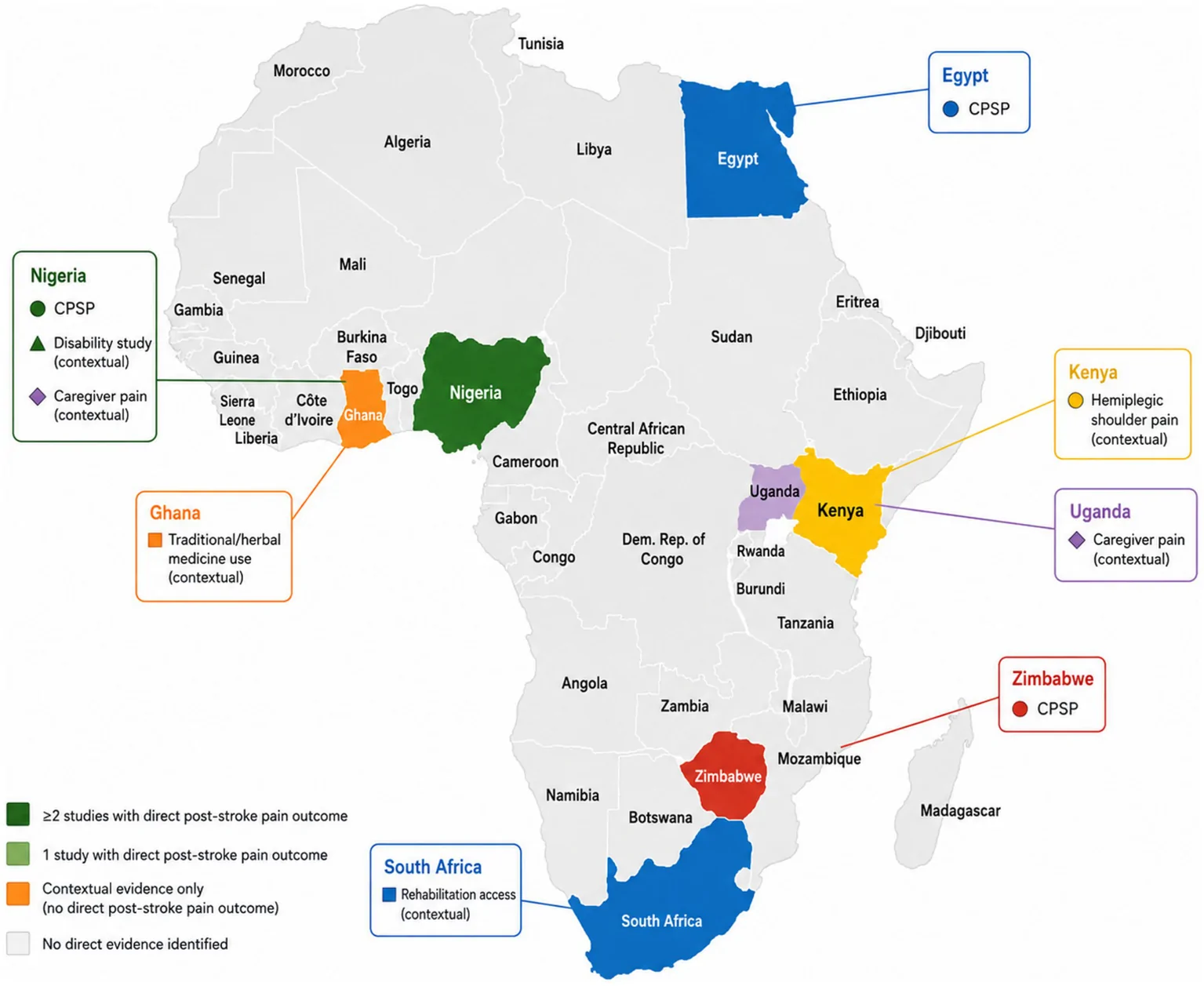
Current evidence from Africa.

This wide variation, from 5% in Kano to 35.4% in Ismailia (roughly a seven-fold difference), likely reflects multiple sources of heterogeneity rather than a demonstrated geographic difference. The Egyptian study combined structured clinical assessment with somatosensory evoked potentials (SSEPs) and MRI lesion correlation, whereas the Nigerian and Zimbabwean cohorts relied primarily on clinical questionnaires or screening approaches (8–10). MRI and SSEPs may support lesion localization and phenotyping, but they are not required to diagnose CPSP: diagnosis remains clinical, based on neuropathic pain characteristics, sensory abnormalities in a neuroanatomically plausible distribution, evidence of a relevant central lesion, and exclusion of competing causes. Differences in case definition, assessment intensity, stroke subtype, sampling, study design, and small single-center samples may all contribute to the observed prevalence spread. A brief critical appraisal is therefore warranted. The Egyptian cohort (*n* = 65) used detailed multimodal characterization but was the smallest and was drawn from a single hospital, increasing the potential for selection bias; its 35.4% estimate should not be treated as population prevalence. The Nigerian cohort (n = 120) used validated neuropathic pain instruments (DN4 and LANSS), but the source paper contains an unresolved discrepancy between its headline 6/120 prevalence and an 11-patient sensory-profile subgroup, limiting confidence in the point estimate (9). The Zimbabwean cohort (*n* = 166) was also a single-center tertiary-clinic sample. None of these studies provides a population-based estimate, and none adequately represents survivors who do not reach tertiary care.

Beyond CPSP, African evidence on other post-stroke pain phenotypes remains limited. A Nigerian study of 121 stroke survivors documented disability but did not specifically evaluate pain interventions (11). In Kenya, a qualitative study of 15 stroke-survivor dyads identified substantial gaps in rehabilitation and orthotic services (12). Nigerian studies have also documented substantial musculoskeletal symptoms among informal caregivers, although these represent caregiver burden rather than post-stroke pain in survivors (13–15).

The country-level distribution of available evidence is itself informative. Published post-stroke pain data cluster in Egypt, Nigeria, Ghana, South Africa, Zimbabwe, and Kenya, mirroring the broader concentration of African stroke research in North, West, and Southern Africa, while large populous regions, including Francophone West and Central Africa, the Horn of Africa, and much of East Africa, remain essentially undocumented for this outcome. A general descriptive account of stroke impact in central Uganda found low attendance at post-discharge rehabilitation and a single neurologist covering the country’s largest referral hospital, but did not report pain outcomes specifically, illustrating how structural constraints can suppress pain data collection even where broader stroke research exists (13, 14). In Morocco, journalistic and clinical commentary describe substantial unaddressed post-stroke neurological sequelae and a growing but unevaluated interest in acupuncture as an adjunct for post-stroke pain and recovery (15, 16), but no peer-reviewed Moroccan prevalence study for any post-stroke pain phenotype was identified. Table 1 summarizes the principal direct African evidence identified for this review, illustrating both the phenotypic focus of existing studies (concentrated on CPSP and shoulder-related pain) and the near-total absence of published data on post-stroke headache, spasticity-related pain quantified independently of global disability scores, and complex regional pain syndrome in African cohorts specifically; Table 2 separately summarizes indirect, contextual evidence (caregiver pain, traditional-medicine use, rehabilitation workforce access) relevant to the broader post-stroke pain landscape but not included in this phenotype synthesis.

**Table 1:** Direct African evidence on post-stroke pain phenotypes.

Country	Design/setting	N	Stroke type	Timing	Pain assessment	Pain primary?	Pain outcome/key finding
Egypt	Prospective cohort; Suez Canal University Hospitals (8)	65	Ischemic	Not reported	Structured questionnaire; SSEP + MRI	Yes	CPSP: 35.4% (23/65)
Nigeria	Cross-sectional; Aminu Kano Teaching Hospital (9)	120	Not reported	50% of CPSP cases within 3 months	DN4; LANSS	Yes	CPSP: 5% (6/120); source numerator inconsistency noted in text
Zimbabwe	Cross-sectional; tertiary stroke clinic (10)	166	Not reported	Not reported	Not specified	Yes	CPSP: 8%; younger age associated
Nigeria	Cross-sectional disability survey; two tertiary institutions in South-Western Nigeria (11)	121	Not specified	At least 3 months after stroke	WHO Disability Assessment Schedule (disability measure; not a dedicated pain tool)	No	Pain-related disability items may be captured within disability assessment; this study did not test a pain intervention.

**Table 2:** Contextual and health-system evidence relevant to post-stroke pain in Africa.

Country	Study design/setting	*N*	Pain assessment tool	Phenotype(s) reported	Key finding
Nigeria	Cross-sectional surveys — Caregiver musculoskeletal symptom surveys, Maiduguri and Kano (39–41)	90; 300; 218, respectively	Nordic Musculoskeletal Questionnaire used in the caregiver studies; caregiver outcomes, not survivor pain	Caregiver musculoskeletal pain	High prevalence of low back and shoulder-region musculoskeletal symptoms among informal caregivers performing manual handling and transfers. Caregiver pain, not a post-stroke pain syndrome in the survivor.
Ghana	Cohort study — Komfo Anokye Teaching Hospital, stroke survivor cohort (33)	106	N/A	Traditional-medicine use	43.4% of 106 stroke survivors reported concurrent traditional herbal medicine use, frequently for expectation of faster motor recovery. Traditional-medicine use, not a direct pain outcome.
Multiple LMICs/Ghana	Scoping review synthesis — Continent-wide and Ghana-specific rehabilitation workforce/access findings (30, 31)	Not reported in source	N/A	Rehabilitation workforce/access	(30) reports fewer than 10 rehabilitation professionals per million population across many low- and middle-income countries generally (not South Africa-specific, as an earlier draft of this row incorrectly implied) (31); an Africa-wide scoping review, reports that an estimated 20% of stroke survivors experienced delayed or absent rehabilitation, and specifically that only 60% of stroke survivors in a Ghanaian study had access to a physiotherapist at all. Rehabilitation workforce access, not a direct pain outcome.
Zimbabwe	Descriptive occupational-therapy perspective; tertiary rehabilitation setting (21)	N/A	N/A	Hemiplegic shoulder pain context	Describes HSP presentation and occupational-therapy management; contextual source, not prevalence evidence.
Kenya	Qualitative rehabilitation-stakeholder study, Eldoret (12)	15 stroke-survivor dyads	N/A	Shoulder subluxation/rehabilitation context	Qualitative study of rehabilitation access/support. The “up to 80%” shoulder-subluxation figure appears in the paper as cited background literature, not as prevalence measured in its 15 participants.

Table 1 is restricted to empirical survivor-level African evidence reporting a direct post-stroke pain outcome.

The concentration of evidence in a small number of countries should be interpreted as an evidence gap, not as evidence that unstudied phenotypes are rare. Global HSP prevalence estimates range from roughly 22% to over 55% depending on population, timing, and case definition (17–20), but the available data do not establish whether African populations have similar, lower, or higher prevalence. Differences in clinical recognition, research priorities, access to follow-up, and use of structured pain assessment may influence what is detected and published. Dedicated, standardized African studies are needed before continent-wide epidemiological inferences can be made.

## 4 Clinical phenotypes

Although African-specific prevalence data now exist for several phenotypes, coverage remains uneven, and understanding each phenotype’s mechanism and presentation is essential to building context-appropriate assessment and management pathways for African settings.

### 4.1 Hemiplegic shoulder pain

Hemiplegic shoulder pain (HSP) is widely reported as the most common musculoskeletal complication after stroke internationally, with incidence estimates of roughly 10 to 22% and prevalence estimates of 22 to 47% in systematic review pooled analyses, though individual cohort studies report figures as high as 55 to 84% depending on timing and population (17–20). Onset typically occurs two to 3 months post-stroke, though it can present as early as 2 weeks (20). Mechanistically, HSP arises from a combination of glenohumeral subluxation secondary to flaccid paralysis, rotator cuff pathology (including supraspinatus tendon thickening and impingement), spasticity and altered scapular positioning, and in some cases a neurogenic contribution from thalamic or central pain pathways, making it as much a biomechanical as a neurological complication (18–20). HSP is consistently associated with reduced activities-of-daily-living performance, longer hospital stays, higher readmission rates, and interference with active rehabilitation participation (17, 19). In the African literature specifically, a Kenyan qualitative study identified major rehabilitation and orthotic gaps relevant to shoulder-subluxation management (12); HSP has also been described from a Zimbabwean occupational therapy perspective addressing presentation and treatment approach in a Southern African rehabilitation setting (21), and shoulder pain features among the common disability domains identified in Nigerian post-stroke disability surveys (11). Dedicated African prevalence studies using validated shoulder-pain outcome measures, however, remain scarce.

### 4.2 Central post-stroke pain

Central post-stroke pain (CPSP) is a central neuropathic pain syndrome arising after a stroke affecting somatosensory pathways. It commonly presents with spontaneous burning, aching, or electric-shock-like pain together with sensory abnormalities such as allodynia, hyperalgesia, dysesthesia, or altered temperature sensation in a distribution compatible with the central lesion. CPSP is fundamentally a clinical diagnosis: neuroimaging establishes the relevant stroke lesion and may support anatomical plausibility, while electrophysiological testing or quantitative sensory testing can assist phenotyping in selected cases but is not required for diagnosis. Three dedicated African prevalence studies report markedly different estimates: 35.4% in a small Egyptian ischemic-stroke cohort, 5% in Nigeria, and 8% in Zimbabwe (8–10). The Nigerian and Zimbabwean estimates lie toward the lower end of international ranges, whereas the Egyptian estimate is high; however, these three single-center studies are too small and methodologically heterogeneous to establish whether African CPSP prevalence differs from global estimates. The appropriate conclusion is therefore that African prevalence remains uncertain and requires larger, multicountry studies using harmonized clinical case definitions and standardized sensory assessment.

### 4.3 Spasticity-related musculoskeletal pain

Spasticity, a velocity-dependent increase in muscle tone arising from upper motor neuron injury, is a common post-stroke complication; reported prevalence varies substantially with stroke subtype, timing, and measurement method (22, 23). Spasticity can contribute to pain through sustained abnormal muscle activity, painful spasms, altered biomechanics, and secondary contracture. Dedicated African studies quantifying spasticity-related pain as a distinct outcome were not identified in this review. The Nigerian disability study cited elsewhere was not designed to establish a spasticity-pain mechanism or treatment effect (11), and the Kenyan qualitative study primarily documents rehabilitation and orthotic access rather than spasticity-related pain prevalence (12).

### 4.4 Post-stroke headache

Post-stroke headache requires distinction by both timing and stroke subtype. The International Classification of Headache Disorders recognizes headache attributed to ischemic stroke, intracerebral hemorrhage (ICH), and subarachnoid hemorrhage (SAH), with acute headache temporally related to the cerebrovascular event and persistent headache continuing after the acute period. These entities should not be treated as a single phenotype because mechanisms, clinical course, and diagnostic implications differ across ischemic stroke, ICH, and SAH. Recent international evidence reinforces this heterogeneity: a 2026 systematic review and meta-analysis of 24 studies (4,688 patients) reported an overall pooled headache prevalence of 46.1% after hemorrhagic stroke, with very high between-study heterogeneity, and separately evaluated acute/subacute, persistent, and severe headache (24). Headache quality may resemble primary headache phenotypes in some survivors, including migraine-like or tension-type-like features, but phenotype reporting is inconsistent and cannot be assumed to differ systematically by stroke subtype without direct data. No dedicated African prevalence study of post-stroke headache was identified in this review, and no African study was found that reliably stratified headache prevalence, timing, or clinical phenotype by ischemic stroke, ICH, or SAH. Hypertension is highly prevalent among African stroke populations, but evidence linking hypertension itself or antihypertensive therapy specifically to post-stroke headache is insufficient; any such association remains inferential and should not be presented as an established post-stroke mechanism. Dedicated prospective African studies should therefore record stroke subtype, headache onset and duration, headache phenotype, medication exposure, and competing secondary causes.

### 4.5 Complex regional pain syndrome/shoulder-hand syndrome

Complex regional pain syndrome (CRPS), sometimes termed shoulder-hand syndrome in the post-stroke context, is characterized by pain, vasomotor and sudomotor changes, edema, and trophic skin changes in the hemiplegic limb, typically the hand and wrist. Diagnosis is based on the Budapest criteria, which require continuing pain disproportionate to the inciting event, with symptoms and signs across sensory, vasomotor, sudomotor/oedema, and motor/trophic domains, and no alternative diagnosis that better explains the presentation. These criteria help distinguish CRPS from hemiplegic shoulder pain, immobility-related oedema, spasticity, and peripheral nerve injury. It is considered a less common but more disabling post-stroke pain phenotype internationally, frequently co-occurring with or being difficult to distinguish from hemiplegic shoulder pain, with evidence-based rehabilitation reviews addressing the two conditions jointly given their overlapping presentation and management (20, 25). No African-specific prevalence or descriptive data for post-stroke CRPS were identified in this search. Its diagnosis depends on distinctive vasomotor and trophic signs, which require a degree of clinical familiarity and examination time not always available in high-volume, understaffed African rehabilitation settings. Whether CRPS is uncommon, under-recognized, or simply understudied in African stroke populations remains unknown.

### 4.6 Other phenotypes

Myofascial pain involving shoulder girdle trigger points has been described as a substantial contributor to poststroke shoulder pain internationally, with targeted trigger-point interventions producing meaningful pain reduction in non-African cohorts (26). Contracture-related pain, arising from prolonged untreated spasticity and immobility, is a plausible and likely underrecognized contributor to chronic post-stroke pain in African settings given the workforce and access constraints on early rehabilitation described in Section 7, though it has not been directly quantified in African populations (Table 3).

**Table 3:** Phenotype-specific clinical summary and African evidence gaps.

Phenotype	Typical timing/course	Key clinical features	Diagnostic approach	African evidence	Priority gap
CPSP	Often develops within weeks to months; may be persistent	Burning/aching/electric pain; allodynia, hyperalgesia, dysesthesia, sensory loss	Clinical diagnosis plus relevant central lesion; structured sensory examination; MRI/SSEP/QST are supportive, not mandatory	Dedicated studies in Egypt, Nigeria, Zimbabwe; estimates 5–35.4% with major heterogeneity	Multicountry prospective studies using harmonized clinical criteria
Hemiplegic shoulder pain	Often emerges in first weeks to months	Shoulder pain with weakness, subluxation, altered biomechanics, soft-tissue pathology and/or spasticity	Clinical musculoskeletal and neurologic assessment; imaging when indicated for competing structural pathology	No dedicated African HSP prevalence study identified in this review; Nigerian disability data and Kenyan/Zimbabwean contextual reports provide indirect evidence.	Validated prospective HSP prevalence and mechanism studies
Spasticity-related pain	May emerge as spasticity develops; chronic pain can accompany contracture	Painful hypertonia/spasms, restricted range of motion, secondary contracture	Clinical examination of tone, range, pain triggers and function	No dedicated African prevalence study identified	Phenotype-specific prevalence and treatment-outcome studies
Post-stroke headache	Acute after stroke or persistent thereafter; course varies by ischemic stroke, ICH and SAH	Headache characteristics vary; migraine-like or tension-type-like features may occur but are inconsistently reported	Classify by stroke subtype, temporal relationship, duration, phenotype and exclusion of other secondary causes	No dedicated African prevalence or subtype-stratified study identified	Prospective studies recording subtype, timing, phenotype and medication exposure
CRPS/shoulder-hand syndrome	Usually develops during post-stroke recovery; may become persistent	Disproportionate pain with sensory, vasomotor, sudomotor/edema and motor/trophic abnormalities	Budapest clinical criteria; exclude HSP, immobility edema, spasticity and peripheral nerve injury	No dedicated African prevalence study identified	Epidemiology and diagnostic-recognition studies using Budapest criteria

## 5 Unmet needs for post stroke pain in Africa

A foundational barrier to characterizing and managing post-stroke pain in Africa is the near-total absence of pain assessment tools developed or validated in African languages and cultural contexts. None of the internationally standard neuropathic pain screening instruments, including the Douleur Neuropathique en 4 Questions (DN4), the Leeds Assessment of Neuropathic Symptoms and Signs (LANSS), and the ID-Pain questionnaire, were developed in an African language, leaving investigators and clinicians working in Africa to either translate existing tools, use them in their language of origin, or develop new instruments altogether (27). This gap is illustrated concretely by the studies reviewed above: both the Nigerian and Zimbabwean CPSP studies relied on DN4 or LANSS in translated or original-language form rather than an instrument developed for the local population (9, 10). Translation is the most commonly used strategy outside Sub-Saharan Africa, and evidence suggests the core symptom constructs underlying these tools remain broadly stable across translated versions; however, individual descriptor terms central to neuropathic pain phenotyping, such as “tingling” or “burning,” do not translate uniformly across languages and cultures, risking reduced content validity even where face validity is preserved (27). This challenge is compounded in African settings by extreme linguistic diversity within single countries. South Africa alone has 11 official languages, making single-tool translation strategies impractical at national scale (27). A broader systematic review of neuropathic pain screening questionnaires similarly found that cross-cultural adaptations generally carry weaker measurement-property evidence than original-language versions, reinforcing that imported tools cannot simply be assumed valid once translated (28).

Beyond language, cultural and idiomatic differences in pain expression present a further assessment barrier. Where local languages lack precise equivalents for neuropathic descriptors, patients may under-report or differently characterize symptoms, and clinicians may have difficulty distinguishing neuropathic from musculoskeletal pain. Structured history and sensory examination are central to this distinction. Neuroimaging is important for confirming and localizing the causal stroke lesion, while quantitative sensory testing or electrophysiological studies may provide additional phenotypic information where available; none should be presented as a mandatory diagnostic test for CPSP. Limited access to specialist assessment and imaging outside many urban tertiary centers may nevertheless complicate exclusion of competing diagnoses and detailed phenotyping (8).

Finally, assessment is constrained by workforce and training gaps. Where stroke follow-up is delivered by generalist physicians, nurses, or overstretched physiotherapy services rather than clinicians specifically trained in post-stroke complications, pain is liable to be treated as an expected, unremarkable consequence of stroke rather than a distinct, potentially treatable syndrome warranting structured evaluation. This mirrors the pattern already described for post-stroke depression and cognitive impairment in Africa, both of which remain under-recognized despite confirmed high prevalence, reinforcing that non-motor post-stroke complications as a category are systematically deprioritized relative to motor and mortality outcomes in routine African stroke care (6, 7).

## 6 Management

### 6.1 Pharmacological management

Pharmacological management should be tailored to the pain phenotype. CPSP and neuropathic components of CRPS require neuropathic pain therapies, whereas HSP and spasticity-related pain are managed with analgesic and antispasticity approaches. Post-stroke headache requires headache-specific treatment tailored to the clinical phenotype and underlying cerebrovascular condition. First-line pharmacological agents for the neuropathic phenotypes, principally neuropathic post-stroke pain internationally include tricyclic antidepressants, gabapentinoids (gabapentin and pregabalin), and serotonin-noradrenaline reuptake inhibitors such as duloxetine (29). In many African health systems, consistent public-sector availability, affordability, and prescriber familiarity with these agents for neuropathic indications specifically (as opposed to their more established use in epilepsy or general psychiatry) cannot be assumed, and formulary access varies considerably between and within countries. Where gabapentinoids are available, cost frequently falls to out-of-pocket payment by patients and families already managing the broader costs of stroke care, a burden compounded by the polypharmacy many stroke survivors already require for secondary prevention (antihypertensives, statins, antiplatelets). For HSP and other musculoskeletal and spasticity-related pain, simple analgesics and oral antispasticity agents such as baclofen are more likely to be accessible, while post-stroke headache is generally managed with standard first-line headache therapy (e.g., simple analgesics, and preventive agents where hypertension or medication-overuse contributions are addressed) rather than the neuropathic-pain regimen described above, but systematic data on any of these phenotype-specific prescribing patterns for post-stroke pain specifically in African settings are lacking.

### 6.2 Non-pharmacological and rehabilitative management

Physical rehabilitation, including appropriate positioning, range-of-motion exercise, task-specific training, and management of biomechanical contributors, is central to prevention and management of hemiplegic shoulder pain and spasticity-related disability internationally. However, the African sources identified in this review provide substantially stronger evidence about rehabilitation access and service constraints than about pain-treatment efficacy. The Nigerian disability study (11), for example, was observational and should not be cited as an intervention demonstrating pain reduction. Workforce constraints are substantial: a 2026 review reports WHO-derived estimates that many LMICs have fewer than 10 rehabilitation professionals per million population and notes severe shortages of speech and language therapy in parts of Sub-Saharan Africa (30). An Africa-focused scoping review found workforce shortages in 31.25% (5/16) of included studies and reported, from underlying South African and Ghanaian studies, delayed or inadequate rehabilitation in 20% of one South African cohort and physiotherapy access in 60% of one Ghanaian cohort (31). These figures are contextual and should not be generalized to Africa as a whole.

### 6.3 Interventional options

Interventional treatment should also be phenotype-specific. Botulinum toxin type A is supported by international trial and meta-analytic evidence for focal post-stroke spasticity, with consistent reduction in muscle tone and some evidence of benefit for associated pain when combined with rehabilitation (32). Intra-articular corticosteroid injection may be considered for selected shoulder pathology, while neuromodulatory approaches are reserved for carefully selected refractory central pain. This review identified no peer-reviewed study quantifying access to botulinum toxin specifically for post-stroke spasticity across African health systems. Rather than asserting continent-wide unavailability, the available evidence indicates that access, affordability, geographic distribution, and public-sector availability of these interventions remain insufficiently characterized and warrant direct study.

### 6.4 Traditional and complementary medicine

Traditional and complementary medicine use is documented among African stroke survivors, but utilization should not be equated with efficacy. A cross-sectional survey of 106 consecutive stroke survivors at a tertiary center in Kumasi, Ghana found that 43.4% reported traditional herbal medicine use; the study examined frequency and reasons for use rather than effectiveness for post-stroke pain (33). Traditional health practitioners are legally recognized in South Africa, but peer-reviewed literature describes integration and collaboration with biomedical services as incomplete and contested rather than fully established (32). Broader literature describes herbal and spiritual dimensions of African traditional medicine (34). A Ghanaian mixed-methods study protocol was designed to compare conventional and complementary rehabilitation pathways but, as a protocol, does not provide treatment-effectiveness results (35). Accordingly, the efficacy, safety, and herb-drug interaction profile of traditional remedies for post-stroke pain remain insufficiently established.

## 7 Structural and health system barriers

The unmet needs described above in assessment and management do not exist in isolation; they are downstream of broader structural constraints on African stroke care. Specialist workforce density is a central limiting factor: several African nations have fewer than one neurologist per million population, compared with approximately 100 per million in high-income countries, and continent-wide estimates place stroke-specialist density at roughly three neurologists per 10 million people compared with up to 900 per 10 million in parts of Europe, a roughly 300-fold disparity (36, 37). Uganda illustrates this concretely: Mulago National Referral Hospital, the country’s largest public tertiary facility with roughly 1,500 beds, has historically operated with a single staff neurologist, with most neurologic care, including post-stroke follow-up, delivered by general internists instead (14). This shortage is compounded by pronounced urban–rural inequity even within countries with relatively better-resourced systems; South Africa’s urban centers report neurologist densities many times higher than rural regions of the same country (37). Post-discharge rehabilitation follow-up is similarly inconsistent: reviews of African stroke rehabilitation services describe an absence of structured post-discharge follow-up in East African settings such as Tanzania and Rwanda, while South Africa offers follow-up services that are available but frequently neither comprehensive nor reaching the majority of survivors who need them (38). Out-of-pocket cost for medications, rehabilitation, and specialist consultation further compounds these access barriers, particularly for the chronic, ongoing care that post-stroke pain management requires rather than a single acute intervention (37). Professional migration out of the region (“brain drain”) perpetuates workforce shortages in a self-reinforcing cycle, as limited training and career-progression opportunities push newly trained specialists toward higher-income health systems (36). Collectively, these structural constraints mean that even where post-stroke pain is correctly identified, the health-system capacity to deliver sustained, appropriate management is frequently absent.

## 8 Research and policy recommendations

Based on the gaps identified in this review, we propose the following priorities for African post-stroke pain research and health-system planning, organized here by implementation horizon rather than as an undifferentiated list. Long-term/foundational research priorities: Phenotype-specific prevalence studies are urgently needed across a wider range of African countries and settings than the current Egypt-Nigeria-Ghana-South Africa-Zimbabwe-Kenya cluster, using standardized, ideally harmonized case definitions and, where feasible, objective adjuncts such as somatosensory evoked potentials or structured sensory examination to allow cross-country comparison and reduce the assessment-driven variability seen in the CPSP literature to date. Post-stroke headache and complex regional pain syndrome in particular have no dedicated African data at all and should be prioritized alongside continued CPSP and HSP characterization, and francophone, Lusophone, and Arabic-speaking regions beyond Egypt remain almost entirely unstudied. Also, the development and rigorous validation of pain assessment tools in major African languages, following robust cross-cultural adaptation methodology rather than simple translation, should be treated as foundational infrastructure for the entire research and clinical agenda described in this review; without valid instruments, prevalence estimates and treatment-outcome studies alike rest on uncertain measurement footing.

*Medium-term/pragmatic priorities*: Pragmatic trials of low-cost, rehabilitation-based interventions, including positioning protocols, caregiver-delivered exercise programs, orthotic provision models such as the gap identified in Kenya, and task-shifted assessment models using trained non-specialist health workers, are more likely to be implementable at scale across African health systems than trials of high-cost interventional or pharmacological options, and should be prioritized accordingly given current workforce constraints. Research directly engaging with traditional and complementary medicine use, rather than treating it as a confounder to be excluded, is needed to characterize both safety (particularly herb-drug interactions relevant to secondary stroke prevention medications) and any genuine symptomatic benefit, given the demonstrated scale of concurrent use among African stroke survivors and the emerging, currently unevaluated interest in practices such as acupuncture in settings like Morocco. *Short-term/low-cost policy priorities*: Integrating structured pain screening into existing post-stroke follow-up pathways, however limited those pathways currently are, represents a comparatively low-cost intervention that could meaningfully improve recognition even in advance of broader workforce expansion. Regional bodies such as the African Stroke Organization are well placed to coordinate harmonized screening protocols and training curricula across member countries.

## 9 Limitations

This review has limitations inherent to both its narrative design and the underlying evidence base. No formal risk-of-bias appraisal or meta-analysis was undertaken. To reduce overinterpretation, we have nevertheless critically considered sample size, single-center sampling, case ascertainment, and reporting inconsistencies when interpreting the principal CPSP studies. Publication and language bias are also likely: although no language filter was applied during searching, only English-language full texts could be assessed, potentially underrepresenting Francophone, Lusophone, and Arabic-language African research. Most direct evidence comes from small hospital-based cohorts, limiting generalizability to community-dwelling and rural survivors. Also, for phenotypes with few or no African studies, including post-stroke headache, CRPS, and spasticity-related pain, absence of published evidence should not be interpreted as evidence of absence or as proof of underdiagnosis; prevalence and recognition remain unknown and require direct study.

## 10 Conclusion

Post-stroke pain in Africa sits at the intersection of two neglected areas of stroke care. Non-motor complications remain systematically under-recognized relative to motor and mortality outcomes, and chronic disease management more broadly is constrained by profound workforce, infrastructure, and access limitations across the continent. The available African studies suggest that post-stroke pain can be substantial where it is actively and rigorously assessed, as in the Egyptian CPSP cohort, but the evidence base remains too limited, heterogeneous, and methodologically uneven to determine whether prevalence differs systematically from global estimates. What can be said with confidence is that recognition, diagnostic capacity, and health-system reach are substantial and addressable gaps, independent of the unresolved epidemiological question. Closing this gap will require coordinated investment in validated, culturally appropriate assessment tools, pragmatic and scalable management models suited to African health-system realities, and a research agenda that takes both the biomedical and traditional dimensions of African stroke care seriously across the continent’s full linguistic and geographic breadth. As survival after stroke continues to improve across Africa, ensuring that the growing population of survivors does not simply exchange mortality risk for undiagnosed, unmanaged chronic pain should be treated as a core priority of African stroke care, not an afterthought.
